# Retraction: Corrigendum: Impact of academic cheating and perceived online learning effectiveness on academic performance during the COVID-19 pandemic among Pakistani students

**DOI:** 10.3389/fpsyg.2025.1689484

**Published:** 2025-09-09

**Authors:** Frontiers Editorial Office

The journal retracts the article cited above.

Following publication, concerns were raised regarding the scientific validity of the article. An investigation was conducted in accordance with Frontiers' policies and it was found that the article contained misleading information that has the potential to mislead readers about previous research and practice related to the topic discussed. Some of the claims in the article are unsupported by the cited literature. The authors failed to provide a satisfactory explanation and as a result, the conclusions of the article have been deemed unreliable and the article has been retracted.

This retraction was approved by the Chief Editors of Frontiers in Psychology and the Chief Executive Editor of Frontiers. The authors do not agree to this retraction.

Frontiers would like to thank the concerned reader who contacted us regarding the published article.

